# Vibration Modes at Terahertz and Infrared Frequencies of Ionic Liquids Consisting of an Imidazolium Cation and a Halogen Anion

**DOI:** 10.3390/ma7117409

**Published:** 2014-11-17

**Authors:** Toshiki Yamada, Yukihiro Tominari, Shukichi Tanaka, Maya Mizuno, Kaori Fukunaga

**Affiliations:** 1 Advanced ICT Research Institute, National Institute of Information and Communications Technology, 588-2 Iwaoka, Kobe 651-2492, Japan; E-Mails: tominari@nict.go.jp (Y.T.); tanakas@nict.go.jp (S.T.); 2 Applied Electromagnetic Research Institute, National Institute of Information and Communications Technology, 4-2-1 Nukuikitamachi, Koganei, Tokyo 184-8795, Japan; E-Mails: mmizuno@nict.go.jp (M.M.); kaori@nict.go.jp (K.F.)

**Keywords:** ionic liquid, imidazolium cation, halogen anion, THz spectroscopy, far-infrared spectroscopy, infrared spectroscopy

## Abstract

The terahertz and infrared frequency vibration modes of room-temperature ionic liquids with imidazolium cations and halogen anions were extensively investigated. There is an intermolecular vibrational mode between the imidazolium ring of an imidazolium cation, a halogen atomic anion with a large absorption coefficient and a broad bandwidth in the low THz frequency region (13–130 cm^−1^), the intramolecular vibrational modes of the alkyl-chain part of an imidazolium cation with a relatively small absorption coefficient in the mid THz frequency region (130–500 cm^−1^), the intramolecular skeletal vibrational modes of an imidazolium ring affected by the interaction between the imidazolium ring, and a halogen anion with a relatively large absorption coefficient in a high THz frequency region (500–670 cm^−1^). Interesting spectroscopic features on the interaction between imidazolium cations and halogen anions was also obtained from spectroscopic studies at IR frequencies (550–3300 cm^−1^). As far as the frequency of the intermolecular vibrational mode is concerned, we found the significance of the reduced mass in determining the intermolecular vibration frequency.

## 1. Introduction

Ionic liquids, which are ambient-temperature molten salts, have been extensively studied because of their unique material properties such as a wide liquid range, extremely low vapor pressure, high electrical conductivity, excellent thermal stability, low combustibility, and excellence as a solvent in catalyzed reactions and the dissociation of biopolymers [1,2,3,4,5]. These properties are expected to have a wide range of applications [6,7,8]. Understanding the intermolecular interactions between cations and anions in ionic liquids is particularly important for clarifying their unique material properties. It is expected that spectroscopic studies related to intra- and/or inter-molecular vibrational modes will play an important role in understanding their nature. So far, the vibrational modes on ionic liquids consisting of an imidazolium cation and a wide range of anionic molecules have been investigated by using THz time-domain spectroscopy (TDS) [9,10,11,12], far-infrared (FIR) [13,14,15], and infrared (IR) spectroscopy [16,17]. In the low THz frequency region, the absorption spectra due to intermolecular vibrations rather than intramolecular vibrations have been observed. However, a systematic study on ionic liquids with imdazolium cations of different alkyl-chain lengths and anionic molecules has not been performed. In IR frequency region, specific vibrational modes of imidazolium ring between 3000 and 3300 cm^−1^ for elucidating the nature of interactions with anionic molecules have been extensively investigated. On the other hand, a systematic study on the vibrational modes of room-temperature ionic liquids that have imidazolium cations and halogen anions as more basic systems has not been performed by using THz, FIR, and IR spectroscopy, although IR spectroscopic studies for some ionic liquids have been performed [16,18,19]. We believe that a detailed spectroscopic characterization in the basic system will be useful benchmark with which to explore the interactions between an imidazolium cation and anionic molecule as more complex system as well as to consider the difference between these systems. In this paper, we extensively studied the vibrational modes of room-temperature ionic liquids that have imidazolium cations with different alkyl-chain lengths and various halogen anions by using TDS, FIR and IR spectroscopy. Since the anion is atom in our study, we did not need to take the vibrational mode or the orientation of the anion into consideration. Therefore many interesting spectroscopic features affected by the interaction between a positively charged imidazolium molecule and a negatively charged halogen atom in liquid states were observed over a wide frequency range (13–3300 cm^−1^). As far as the intermolecular vibrational mode found in the low THz frequency region is concerned, we found that the reduced mass (μ) calculated according to the mass of the methyl-imidazolium ring (the cation) and the mass of the halogen anion, and the force constant (*k*) play an important role in determining the central frequency of the inter-molecular vibration.

## 2. Materials and Methods

THz time-domain spectroscopy (THz-TDS) was performed to measure the physical properties of each sample, such as the frequency-dependent complex dielectric constant and the complex refractive index, by comparing the temporal THz fields both with and without the sample. The THz-TDS spectrometer (Tochigi Nikon, Rayfact RS-01020, Tochigi, Japan) has a maximum band-width from 0.4 to 3.5 THz (13 to 117 cm^−1^). A Fourier transform far-infrared spectroscopic apparatus (JASCO Corporation, VIR-F, Tokyo, Japan) with a wide THz frequency range (30–670 cm^−1^) was equipped with a deuterated l-alanine doped triglycine sulfate (DLATGS) detector, a polyethylene window, and a mercury lamp as the light source. These apparatuses enabled us to perform THz-TDS and far-infrared spectroscopy (FIR) [20]. A Fourier transform infrared spectroscopic apparatus (HORIBA, Ltd., FT-720, Kyoto, Japan) equipped with an attenuated total reflection (ATR) unit (Smiths detection, DuraScope^TM^, London, UK) with a ZnSe crystal was used for IR spectroscopy with a frequency range from 550 to 3300 cm^−1^.

**Figure 1:**
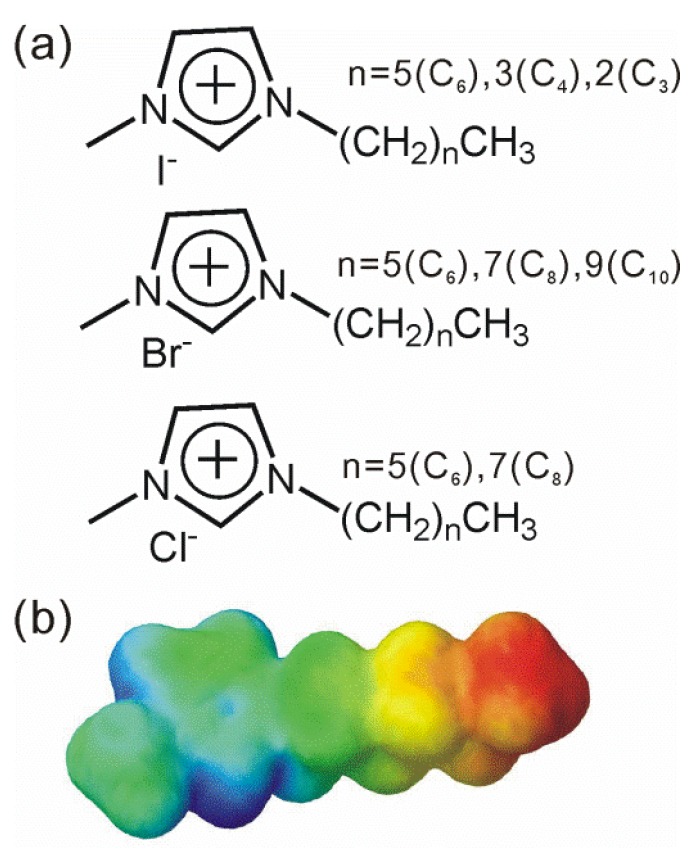
(**a**) Ionic liquids used in our study; (**b**) Calculated electrostatic potential map of a hexyl-metyl-imidazolium cation [C_6_mim^+^]. The most positively charged part is expressed as a deep blue color.

The samples we used were room-temperature ionic liquids, consisting of a series of alkyl-methyl-imidazolium cations and halogen anions, as shown in Figure 1. All the highly pure (>98%) ionic liquids were purchased from Kanto Chemical Co., Tokyo, Japan. Of special note, ionic liquids with iodine anion (I^−^) with a highly purity (>99%) are colorless and very transparent. Hereafter we will use the abbreviated notation for the ionic liquid, such as [C_6_mim^+^][I^−^], where C_6_ and mim stand for the carbon number of the alkyl-chain part and the methyl-imidazolium ring part of the molecular cation, respectively. An ionic liquid sample was put into an assembled cell when the THz-TDS and FIR measurements were conducted. Two 2.0-mm-thick plastic plates consisting of a cyclo-olefin polymer (ZEON Co., Zeonex 480R, Tokyo, Japan), which has a favorable transmittance and a refractive index over a wide THz frequency range, were used as cell windows. The sample thickness was controlled by a spacer, typically 60 μm thick, sandwiched between two plastic windows. Reference measurements without an ionic liquid were performed on the condition that the surfaces of the two plastic plates were in contact. A droplet of an ionic liquid sample was directly used for the ATR-IR measurements. A density functional theory (DFT) calculation was performed for the alkyl-methyl-imidazolium cations. The geometry was optimized at the B3LYP/6-31G(d) level of theory and then the infrared vibrational spectra and electrostatic potential maps were calculated at the same level of theory.

## 3. Results and Discussion

**Figure 2:**
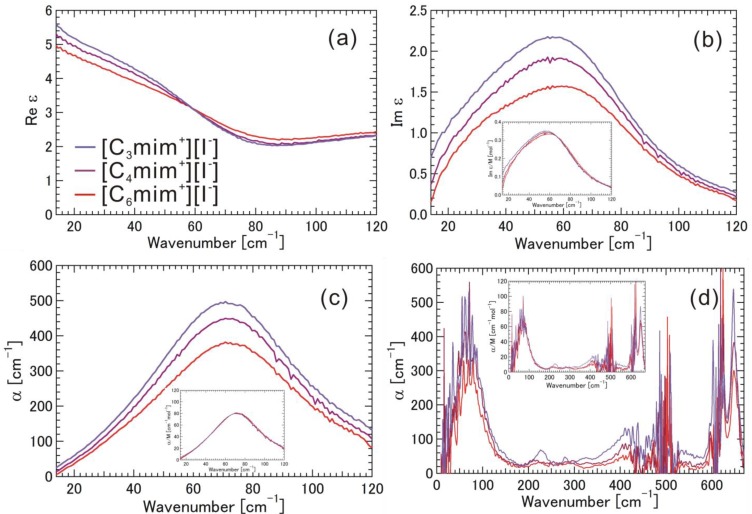
(**a**) Real part of the complex dielectric spectra (Re ε); (**b**) Imaginary part of the complex dielectric spectra (Im ε); (**c**) Absorption coefficients (α) obtained by THz time-domain spectroscopy (THz-TDS); and (**d**) Absorption coefficients (α) obtained by far-infrared (FIR) for the ionic liquids [C_6_mim^+^][I^−^], [C_4_mim^+^][I^−^], and [C_3_mim^+^][I^−^]. The imaginary part of the complex dielectric spectra (Im ε/M) and the absorption coefficient (α/M) normalized by molar concentration are also shown in the insets.

Figure 2a–d shows the complex dielectric spectra (Re ε and Im ε) and the absorption coefficients (α) for the [C_6_mim^+^][I^−^], [C_4_mim^+^][I^−^], and [C_3_mim^+^][I^−^] obtained from both THz-TDS and FIR. The imaginary part of the dielectric constant (Im ε/M) and the absorption coefficient (α/M) normalized by the molar concentration are shown in the insets in Figure 2b–d (see also Figure S1). Im ε/M and α/M were obtained by considering the density of the ionic liquids at room temperature. In the low THz frequency region (13–130 cm^−1^), it was striking that the Im ε/M and α/M spectra were almost the same for [C_6_mim^+^][I^−^], [C_4_mim^+^][I^−^], and [C_3_mim^+^][I^−^]. A relatively large and broad band with a peak at 72.5 cm^−1^ was found in the α/M spectrum in the low THz frequency region. The data indicate that Im ε/M and α/M spectra are not affected by any kind of intramolecular vibration of the alkyl-chain parts of imidazolium cations. According to the calculation of the vibration spectra of imidazolium cations, some intramolecular vibrational modes have weak intensity as a motion of the whole imidazolium cation, including the alkyl-chain part, in the low THz frequency region, and therefore the calculated frequency and intensity of these modes depend on the alkyl-chain length (see Figure S2). It is difficult to explain the relatively large and broad absorption band with same frequencies and intensities in the α/M spectra in the context of only the intramolecular vibrational modes of the imidazolium cations, so it is preferable to explain the absorption band by the number of pairs, or more likely by the electrostatic interaction between methyl-imidazolium ring [mim^+^] with a positive charge, as seen in the electro static potential map in Figure 1b, and the halogen anion [I^−^]. In the mid THz frequency region (130–500 cm^−1^), there were weak but discernible absorption bands in α (α/M) spectra at 225, 290, and ~425 cm^−1^ for [C_6_mim^+^][I^−^]; 210, 290 and ~425 cm^−1^ for [C_4_mim^+^][I^−^]; and 230, 280, 325, and ~425 cm^−1^ for [C_3_mim^+^][I^−^]. According to our calculations, some vibrations have week intensity, including the accordion-like motions of alkyl-chain parts, and the vibrational frequencies differ to some extent depending on the alkyl-chain length (see Figure S2). In particular, we found consistency between the calculation and the experiment for the smallest imidazolium cation [C_3_mim^+^]. Therefore, it is reasonable for these bands to be attributed the intramolecular vibrations of alkyl-chain parts with accordion-like motions. In the high THz frequency region (500–670 cm^−1^), two relatively strong absorption bands at the same frequencies (616 and 647 cm^−1^) were found for these ionic liquids. The peak position of 616 cm^−1^ was determined using the data from the ATR-FTIR spectra described later, because the peak position could not be identified due to the low signal-to-noise ratio in the region (590–630 cm^−1^) of the FIR spectra. These absorption bands inherently originate from the intramolecular vibrational modes of the skeletal vibration of the imidazolium ring, according to our calculations. Therefore, these two absorption bands are almost unaffected by the alkyl-chain length, which was confirmed by our calculations and experiments. In Figure 2a, we found that the magnitude of Re ε in the low-frequency region clearly follows the trend, [C_3_mim^+^][I^−^] > [C_4_mim^+^][I^−^] > [C_6_mim^+^][I^−^], meaning that the electric susceptibility, which depends on the number density and polarizability of the material, follows the same trend in the frequency region. The behavior of Im ε below 30 cm^−1^ in Figure 2b indicates the existence of a dielectric relaxation mode or librational motion of the cations [21,22] for these ionic liquids.

**Figure 3:**
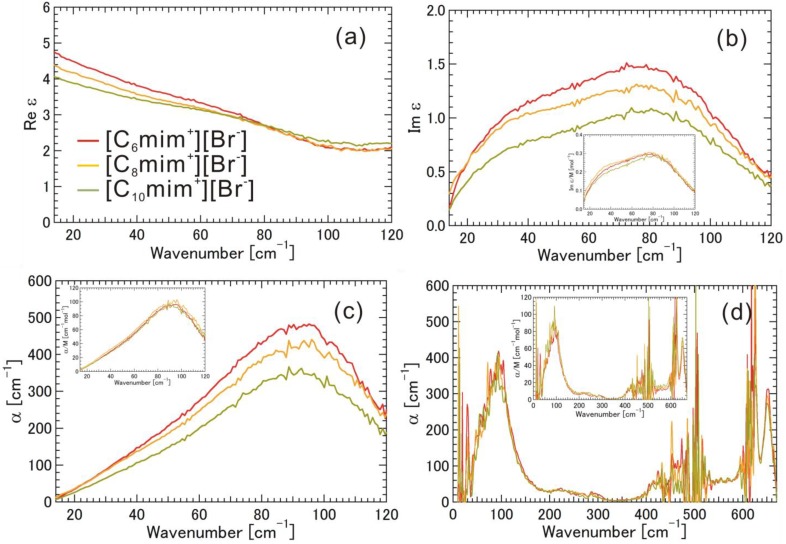
(**a**) Real part of the complex dielectric spectra (Re ε); (**b**) Imaginary part of the complex dielectric spectra (Im ε); (**c**) Absorption coefficients (α) obtained by THz-TDS, and (**d**) Absorption coefficients (α) obtained by FIR, for the ionic liquids [C_6_mim^+^][Br^−^], [C_8_mim^+^][Br^−^], and [C_10_mim^+^][Br^−^]. The imaginary part of the complex dielectric spectra (Im ε/M) and absorption coefficient (α/M) normalized by molar concentration are also shown in the insets.

Figure 3a–d and the insets show Re ε, Im ε (Im ε/M), and α (α/M) spectra for [C_6_mim^+^][Br^−^], [C_8_mim^+^][Br^−^], and [C_10_mim^+^][Br^−^] (see also Figure S3). In the low THz frequency region, Im ε/M and α/M spectra were almost the same for [C_6_mim^+^][Br^−^], [C_8_mim^+^][Br^−^], and [C_10_mim^+^][Br^−^]. A relatively large and broad band with a peak at 92 cm^−1^ in the α (α/M) spectrum was found in the low THz frequency region. Similarly, it is preferable to explain the absorption band according to the electrostatic interaction between [mim^+^] and [Br^−^]. In the mid THz frequency region, there were weak but discernible absorption bands due to the intramolecular vibrations of the alkyl-chain parts in α (α/M) spectra at 225, 290, and ~425 cm^−1^ for [C_6_mim^+^][Br^−^]; 215, 290, and ~425 cm^−1^ for [C_8_mim^+^][Br^−^]; and 215, 290 and ~425 cm^−1^ for [C_10_mim^+^][Br^−^]. In the high THz frequency region, two relatively strong absorption bands were found due to the intramolecular vibrational modes of the skeletal vibration of the imidazolium ring at the same frequencies (620 and 650 cm^−1^) for these ionic liquids. The magnitude of Re ε in the low-frequency region follows the trend, [C_6_mim^+^][Br^−^] > [C_8_mim^+^][Br^−^] > [C_10_mim^+^][Br^−^], and the behavior of Im ε below 30 cm^−1^ indicates the existence of a dielectric relaxation mode or librational motion for these ionic liquids. These features are similar to the cases of ionic liquids with [I^−^].

**Figure 4:**
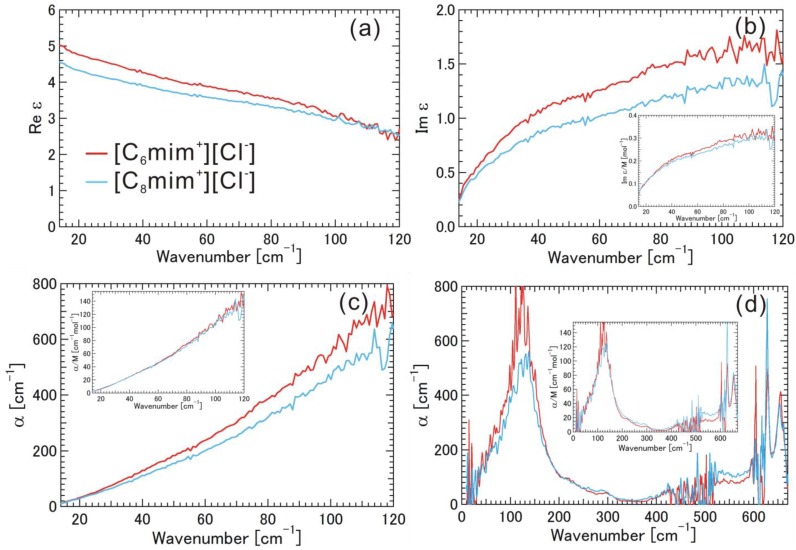
(**a**) Real part of the complex dielectric spectra (Re ε); (**b**) Imaginary part of the complex dielectric spectra (Im ε); (**c**) Absorption coefficients (α) obtained by THz-TDS; and (**d**) Absorption coefficients (α) obtained by FIR, for the ionic liquids [C_6_mim^+^][Cl^−^], [C_8_mim^+^][Cl^−^]. The imaginary part of the complex dielectric spectra (Im ε/M) and the absorption coefficient (α/M) normalized by molar concentration are also shown in the insets.

Figure 4a–d and the insets show Re ε, Im ε (Im ε/M), and α (α/M) spectra for [C_6_mim^+^][Cl^−^], [C_8_mim^+^][Cl^−^] (see also Figure S4). In the low THz frequency region, Im ε/M and α/M spectra were almost the same for [C_6_mim^+^][Cl^−^] and [C_8_mim^+^][Cl^−^]. There was a large and broad band with a peak at 122 cm^−1^ in the α (α/M) spectrum in the low THz frequency region. It is preferable to explain the absorption band according to the electrostatic interaction between [mim^+^] and [Cl^−^]. In the mid THz frequency region, there were weak but discernible absorption bands due to intra-molecular vibrations of alkyl-chain parts in a/M spectra at 225, 290, and ~425 cm^−1^ for [C_6_mim^+^][Cl^−^], and 290 and ~425 cm^−1^ for [C_8_mim^+^][Cl^−^], superimposed on the tail of the large and broad band. In the high THz frequency region, two relatively strong absorption bands were found due to the intramolecular vibrational modes of the skeletal vibration of the imidazolium ring at the same frequencies (628 and 655 cm^−1^) for these ionic liquids. The magnitude of Re ε in the low-frequency region follows the trend, [C_6_mim^+^][Cl^−^] > [C_8_mim^+^][Cl^−^], and the behavior of Im ε below 30 cm^−1^ indicates the existence of a dielectric relaxation mode or librational motion.

**Figure 5:**
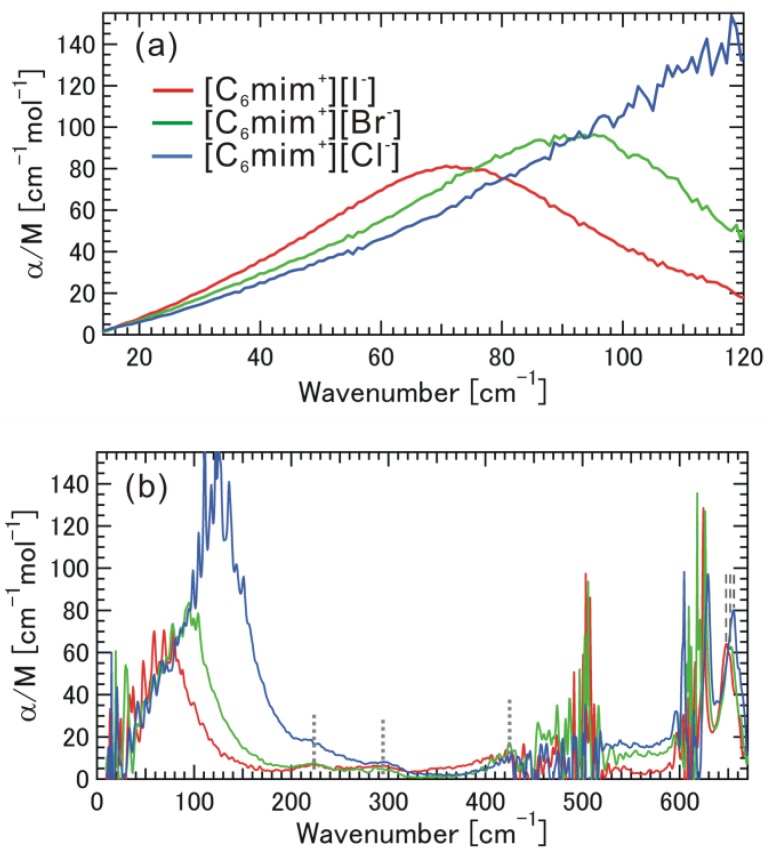
Absorption coefficients (α/M) normalized by molar concentration obtained from (**a**) THz-TDS and (**b**) FIR, for the ionic liquid [C_6_mim^+^][I^−^], [C_6_mim^+^][Br^−^], and [C_6_mim^+^][Cl^−^].

Figure 5a,b shows α/M spectra at 13–670 cm^−1^ for [C_6_mim^+^][I^−^], [C_6_mim^+^][Br^−^], and [C_6_mim^+^][Cl^−^]. In the low THz frequency region (13–130 cm^−1^), strong and broad absorption bands were found at 72.5, 92 and 122 cm^−1^ for [C_6_mim^+^][I^−^], [C_6_mim^+^][Br^−^], and [C_6_mim^+^][Cl^−^]. The ionic liquid with a heavier halogen anion exhibited absorption at a lower frequency, while the ionic liquid with a lighter halogen anion exhibited stronger absorption at a higher frequency. In the mid THz frequency region (130–500 cm^−1^), we can also recognize the intramolecular vibrations due to the accordion-like motions of alkyl-chain parts at almost the same frequencies. Such intramolecular vibrations in ionic liquids have not been identified so far. In the high THz frequency region, the two relatively strong absorption bands due to the intra-molecular vibrational modes of the skeletal vibration of the imidazolium ring clearly are frequency-dependent on the halogen anion species.

Yamamoto *et al.* [10] measured the complex dielectric spectra of imidazolium-based ionic liquids consisting of two kinds of anion and cation molecules using TDS-THz spectroscopy, and pointed out the significance of the interion vibrations. Although we used other imidazolium-based ionic liquids with halogen atomic anions, this statement is in agreement with our interpretation. Furthermore, we systematically confirmed that Im ε/M and Im α/M spectra in low THz frequency regions are independent of the cation species of various alkyl-chain lengths as long as the halogen anion is the same. This is in contrast to the fatty acid system, where the dielectric modes shift toward higher frequencies when increasing the alkyl-chain length [23]. Ludwig *et al*. [11,13,14] extensively investigated the imidazolium-based ionic liquids with a series of anion molecules except for halogen atomic anions by using FIR between 2 and 300 cm^−1^, and FTIR between 3000 and 3300 cm^−1^ in conjunction with DFT calculations. They found the presence of local and directional hydrogen bonding between a specific part of the imidazolium ring and anion molecules with specific configurations and orientations, and the positions of the peak maxima for imidazolium-based ionic liquids with various anion molecules in the low THz frequency region are mainly determined by the force constant *k* from the viewpoint of the simple and fundamental relation ω = (*k*/μ)^1/2^.

**Figure 6:**
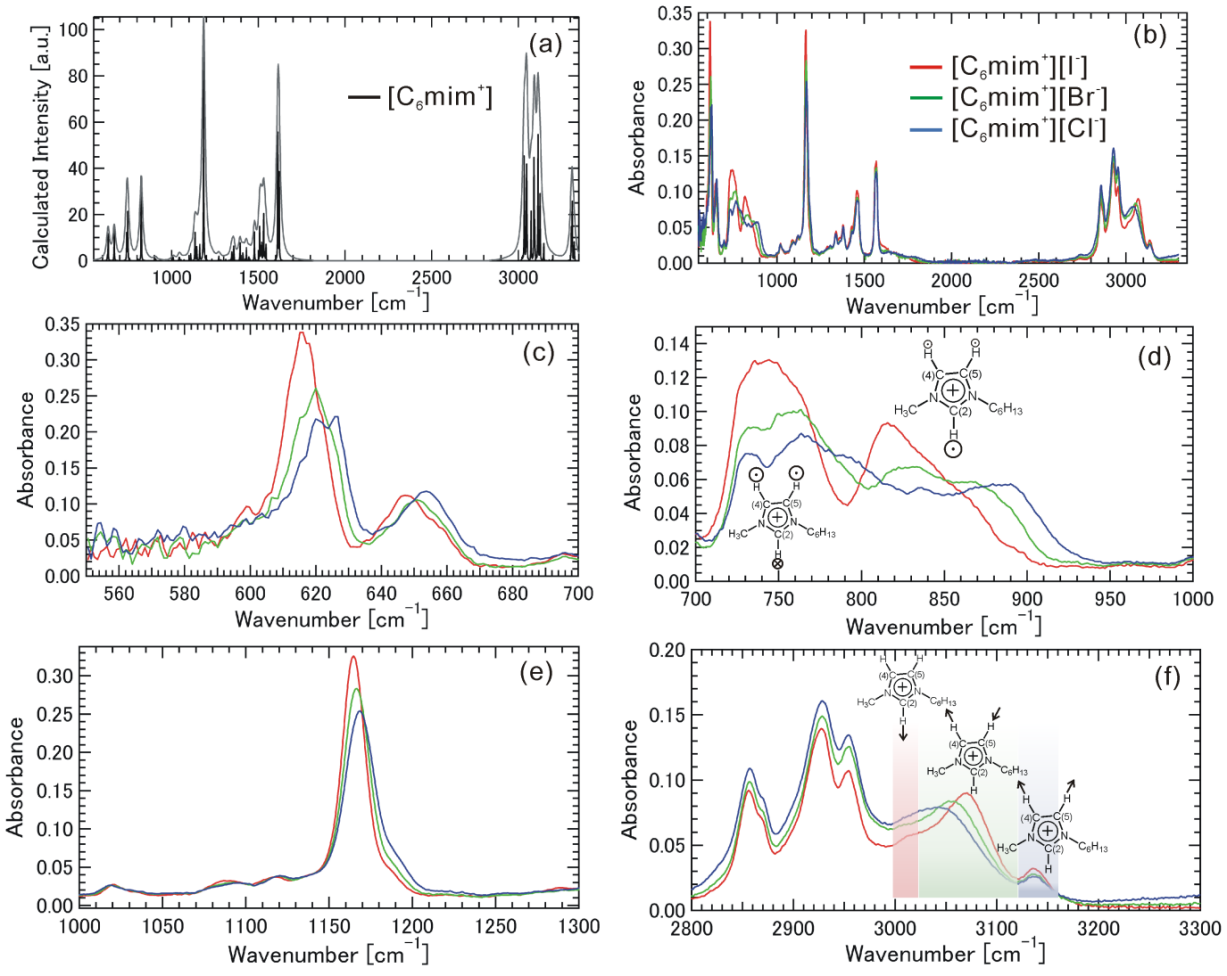
Calculated IR spectrum of [C_6_mim^+^] (**a**) from 550 to 3300 cm^−1^, and experimental IR spectra of [C_6_mim^+^][I^−^], [C_6_mim^+^][Br^−^], and [C_6_mim^+^][Cl^−^]; (**b**) from 550 to 3300 cm^−1^; (**c**) from 550 to 700 cm^−1^; (**d**) from 700 to 1000 cm^−1^; (**e**) from 1000 to 1300 cm^−1^; and (**f**) from 2800 to 3300 cm^−1^. In (**d**), two skeletal vibrations due to the main motions of “H”s in C(2)–H, C(4)–H and C(5)–H perpendicular to the imidazolium ring plane were schematically illustrated, where the circles and their sizes located near “H”s stand for the direction and magnitude of the motions. In (**f**), three vibrational modes in the region between 3000 and 3300 cm^−1^ are also illustrated schematically.

Figure 6a–f shows the ATR-FTIR spectra for [C_6_mim^+^][I^−^], [C_6_mim^+^][Br^−^], and [C_6_mim^+^][Cl^−^], as well as the calculated IR spectrum of [C_6_mim^+^] from 550 to 3300 cm^−1^. The absorption coefficient cannot be well defined due to the ATR geometry, and therefore the IR spectra are not normalized by molar concentration. The calculated IR spectrum in Figure 6a can qualitatively explain the experimental IR spectra in Figure 6b. The interaction between [C_6_mim^+^] and the halogen anion can be deduced by analyzing the IR spectra, because the halogen anion itself does not have a vibrational mode.

The relatively strong peaks between 550 and 700 cm^−1^ in Figure 6c are attributable to the skeletal vibrations of the imidazolium ring. The motions of these skeletal vibrations are partly restricted by the interactions with the halogen anions, and the interactions with the heavier halogen anion leads to a red shift in the peaks. The relatively strong peaks between 700 and 1000 cm^−1^ in Figure 6d are also attributable to the skeletal vibrations of the imidazolium ring. A similar tendency in the data in Figure 6c was observed, although the degree of the red shift due to the interaction is much stronger. According to the calculation (see Figure S5), the skeletal vibrations due to the main motions of “H”s in C(2)–H, C(4)–H and C(5)–H perpendicular to the imidazolium ring plane, which were schematically illustrated in Figure 6d, have relatively strong absorption intensity. Therefore, the data in Figure 6d indicates that such motions are strongly restricted by the interactions with the heavier halogen anions. The strong peak between 1000 and 1300 cm^−1^ in Figure 6e is attributable to the skeletal vibration of the imidazolium ring along with the main-chain direction accompanied by the vibration of methylene groups. Similarly, a red shift was also found, although the degree of this shift is smaller. Thus, the various vibrational modes related to the skeletal vibration of the imidazolium ring in Figure 6c–e is affected by the interactions with the halogen anion, and the interaction with the heavier halogen anion certainly leads to the red shift in the peaks. The degree of the red shift depends on the degree of the coupling of halogen anions to each vibrational mode. In Figure 6f, the several peaks found between 2800 and 3000 cm^−1^ are attributable to typical symmetric and asymmetric stretching vibrations of methylene and methyl groups in the alkyl-chain part. The frequencies of these vibration modes are not affected by the species of halogen anion, indicating that the alkyl-chain part does not have a specific interaction with the halogen anions. We focused our attention on the region between 3000 and 3300 cm^−1^. The significance of the vibrational modes in this region has been pointed out in discussing the interaction between the imidazolium cation and the anion [16,17,24,25]. We found a shoulder structure at around 3015 cm^−1^, a halogen anion interaction-dependent peak between 3040 and 3070 cm^−1^, and a peak at around 3135 cm^−1^. Ludwig *et al*. point out the significance of the frequency of the ^+^C(2)–H stretching vibration in the imidazolium ring due to the interaction of anion molecule (A_molecule_^−^), because this frequency is determined by the balance between ^+^C(2)–H covalent bonding and the H···A_molecule_^−^ interaction [17]. They attributed the nature of ^+^C(2)–H···A_molecule_^−^ interaction as a local and directional hydrogen bonding. Johnson *et al*. [26] point out the significance of local assembly motifs and the limited role of hydrogen bonding in considering ^+^C(2)–H stretching vibration. In our case with the interaction of the halogen anion (A_halogen_^−^); that is, the ^+^C(2)–H stretching vibrational mode is located at around 3015 cm^−1^ as a shoulder structure, as Kim *et al.* [16] also attributed in the case of [C_4_mim^+^][I^−^]. Comparing ^+^C(2)–H···A_halogen_^−^ with ^+^C(2)–H···A_molecule_^−^, the frequency of the mode is strongly red-shifted, indicating that the interaction H···A_halogen_^−^ is inherently stronger than the interaction H···A_molecule_^−^. On the other hand, the frequency shift of the mode among halogen anions (I^−^, Br^−^, and Cl^−^) is small. A peak at around 3135 cm^−1^ is attributable to C(4)–H and C(5)–H stretching vibrations with symmetrical movements to each other. The frequency shift of the mode among halogen anions (I^−^, Br^−^, and Cl^−^) is small. A peak between 3040 and 3070 cm^−1^ is attributable to C(4)–H and C(5)–H stretching vibrations with asymmetrical movements to each other. It is known that this mode is sensitively affected by the position of a halogen anion near ^+^C(2) [16]. A blue shift of this mode among halogen anions in the order of I^−^, Br^−^, and Cl^−^ was observed, indicating that the positions of halogen anions near ^+^C(2) are slightly different, which may be related to the large ionic radii of halogen anions. The frequencies of these C(4)–H and C(5)–H stretching vibrational modes in imidazolium-based ionic liquids with halogen anion are also red-shifted, compared with those with anion molecules [16,17]. The IR data between 2800 and 3300 cm^−1^ show that the interaction H···A_halogen_^−^ in the interactions ^+^C(2)–H···A_halogen_^−^ is inherently strong for all I^−^, Br^−^, and Cl^−^; that is, a halogen anion have the interaction in the vicinity of ^+^C(2) as space-time averages, and the vibrational modes in the alkyl-chain part are not affected by the interaction with halogen anions.

The interaction energies between the imidazolium cations and anions of various ionic liquids were calculated using *ab initio* and DFT calculations [13,27,28,29]. If the anion is a molecule, its typical interaction energies are about 75−90 kcal/mol for ion pairs with specific configurations and orientations [27]. The average interaction energies obtained by considering the small cluster of ion pairs are about 40−55 kcal/mol [13]. If the anion is a halogen anion, its typical interaction energies are about 80−150 kcal/mol [28,29], depending on the calculation level. It is likely that the interaction energies between imidazolium cations and halogen anions are stronger than those between imidazolium cations and molecular anions. On the other hand, we are aware that the structures of ionic liquids are determined by the balance between the complicated packing and screening [30] and, especially, the latter effect, that is, electrostatic interactions that decay slowly over a distance were not considered in these calculations.

Here, we again discuss the data in Figure 2, Figure 3, Figure 4 and Figure 5. Since the absorption frequency does not depend on the alkyl-chain length of the imidazolium cation as shown in Figure 2, Figure 3 and Figure 4 as long as the halogen anion is the same, we considered the effective mass of the cation for the intermolecular vibration to be the mass (*m* = 83) of the methyl-imidazolium ring part [mim^+^]. Considering the fact that the motions of the intramolecuar skeletal vibrations of [mim^+^] are affected by the interaction with halogen anions and the various vibrations of the alkyl-chain parts are hardly affected, as found in the mid and high THz and IR frequency regions, this idea seems to be valid. The electrostatic potential map of the cation as shown in Figure 1b also supports this idea. As far as the anions are concerned, the masses are 126.9 for [I^−^], 79.96 for [Br^−^], and 35.45 for [Cl^−^]. The reduced mass μ is calculated as 1/μ = 1/_μ[mim^+^]_ + 1/_μ[halogen anion^−^]_. Table 1 summarizes their values, where the wavenumber of the absorption peak and the vibrational energy are also added. We noted the significant contribution of the reduced mass to the vibrational frequency or energy.

**Table 1:** Reduced mass μ, $1/\sqrt{\mu }$, wavenumber of absorption peak (cm^−1^), vibration energy (meV) for [mim^+^][I^−^], [mim^+^][Br^−^], [mim^+^][Cl^−^].

Ion pair	Reduced mass μ	$1/\sqrt{\mu }$	Wavenumber (cm^−1^)	Energy (meV)
[mim^+^][I^−^]	50.2	0.14	72.5	9.0
[mim^+^][Br^−^]	40.8	0.16	92	11.4
[mim^+^][Cl^−^]	24.9	0.2	122	15.1

In the case of an imidazolium ionic liquid containing a molecular anion, the significant contribution of the force constant for the frequency shifts in the intermolecular vibrational bands is pointed out [14,17]. In the imidazolium ionic liquid containing Bis(trifluoromethylsulfonyl)imide [NTf_2_^−^] with a mass of 280 and [C_2_mim^+^] with a mass of 97, the absorption band at 97 cm^−1^ was observed [14]. The reduced mass μ is 72 if one assumes entire masses of cation and anion for the intermolecular vibration [14]. The absorption band of [C_2_mim^+^][NTf_2_^−^] at 97 cm^−1^ is close to the absorption band of [C_6_mim^+^][Br^−^] at 92 cm^−1^. We considered the reduced mass μ of [mim^+^][Br^−^] as 40.8. This leads to the larger force constant *k* in [C_2_mim^+^][NTf_2_^−^], compared with *k* in [mim^+^][Br^−^]. Considering the interaction energies in both systems, it seems unlikely. One possibility to solve the discrepancy is to consider the effective masses both for cation and anion involved in the intermolecular vibration, because the presence of localized and directional hydrogen bonding in [C_2_mim^+^][NTf_2_^−^] was shown [17]. However, further discussion on this system is beyond the scope of this paper. In the case of a protic ionic liquid [15,31], the significant contributions both of the force constant and reduced mass for the frequency shifts in the intermolecular vibrational bands is pointed out. They tried to dissert the frequency shifts resulting from reduced masses and force constants by using molecular anions and isotropically substituted cations in protic ionic liquids, and found that both effects contribute to same extend [31]. Thus the contributions of the force constant and reduced mass to the frequency shifts in the intermolecular vibrational band may be dependent on its molecular systems. We noted that the significant contribution of the reduced mass to the frequency shifts as shown in Table 1. On the other hand, we recognize that the estimation of the effective mass of the cation is phenomenological to some extent. If the effective mass of the cation in actual systems is different, the contribution of the force constant to the frequency shifts also appears.

Far infrared spectroscopic studies on tetra-alkylammonium halide crystals and their theoretical analysis point out the significance of reduced mass in determining the intermolecular vibration frequency [32].

The application of other spectroscopic techniques such as Raman spectroscopy [16,33], optical Kerr-effect spectroscopy [20,34] and dielectric relaxation spectroscopy [19,21] to room-temperature ionic liquids consisting of an imidazolium cation and a halogen anion would be very significant, providing us with complementary structural information as well as information on dynamical properties at lower frequencies.

## 4. Conclusions

In conclusion, many interesting intramolecular and intermolecular vibrational modes affected by the interplay between positively charged imidazolium molecules and negatively charged halogen atom in the liquid states were found over a wide frequency range. The data provide us with the microscopic picture of the interaction between a specific part of the imidazolium ring and the halogen anion and the little interaction with the alkyl-chain part of the imidazolium cation. In these systems, we found the significance of the reduced mass in determining the intermolecular vibration frequency. Our systematic data and analysis for ionic liquids consisting of an imidazolium cation and a halogen anion could be useful in elucidating the nature of ionic liquids with anion molecules as more complex systems.

## Supplementary Materials

Supplementary materials can be accessed at: http://www.mdpi.com/1996-1944/7/11/7409/s1.
