# Supplementary material for: Vibration Modes at Terahertz and Infrared Frequencies of Ionic Liquids Consisting of an Imidazolium Cation and a Halogen Anion
Source: Materials (Basel). 2014 Nov 17;7(11):7409–22. doi: 10.3390/ma7117409 (PMC5512643; doi:10.3390/ma7117409)

Supplementary Materials

Figure S1. (**a**) Real part of the complex dielectric spectra (Re ε); (**b**) Imaginary part of the complex dielectric spectra (Im ε/M) normalized by molar concentration; (**c**) Absorption coefficients (α/M) normalized by molar concentration, obtained by THz-TDS; and (**d**) Absorption coefficients (α/M) normalized by molar concentration, obtained by FIR for the ionic liquids $[C_6mim^+][I^-]$, $[C_4mim^+][I^-]$, and $[C_3mim^+][I^-]$.

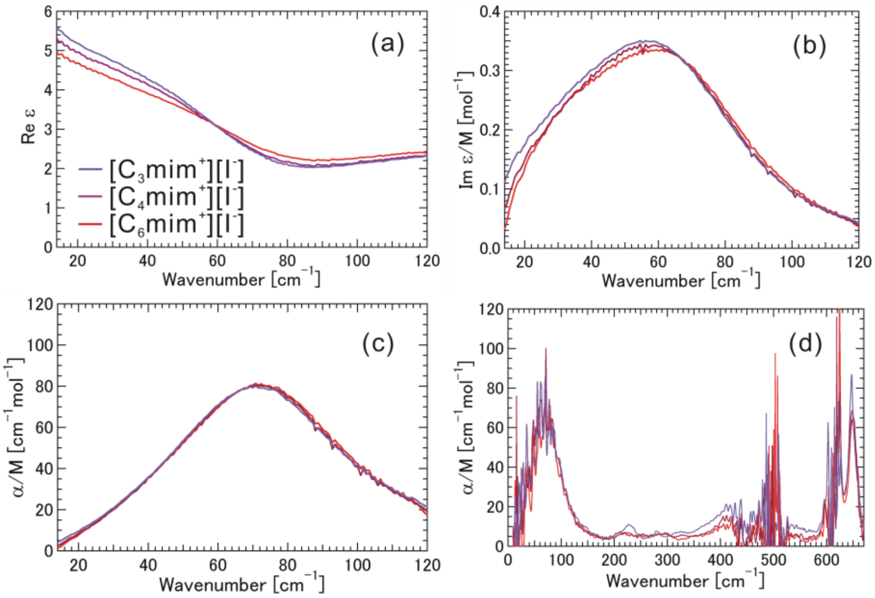

Figure S2. Calculated vibrational spectra for the alkyl-methyl-imidazolium cations (C_3mim^+, C_4mim^+, C_6mim^+, C_8mim^+, and $C_{10}mim^+$) in the low, mid and high THz frequency regions. Density functional theory (DFT) calculations were performed. The geometry was optimized at the B3LYP/6-31G(d) level of theory with a charge of +1 and a multiplicity of singlet, and then the infra-red vibrational spectra were calculated at the same level of theory. The absorption bandwidth was set at 10 cm^{-1} for all absorption bands to make them easy to see.

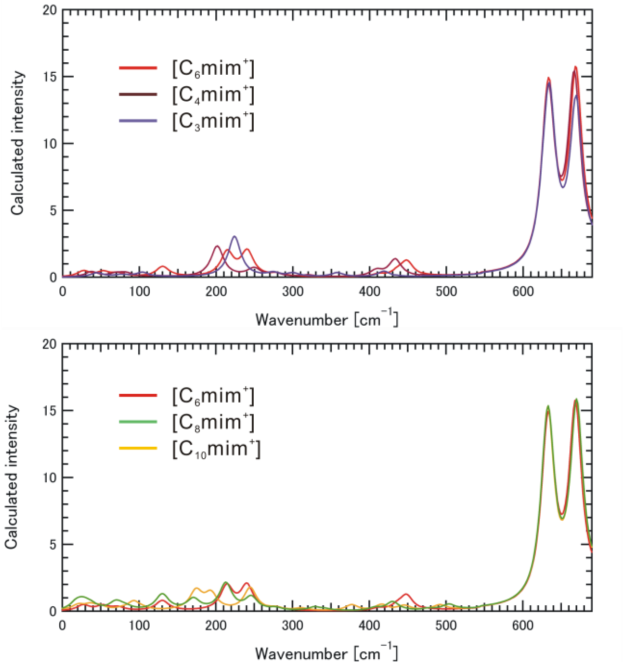

Figure S3. (**a**) Real part of the complex dielectric spectra (Re ε); (**b**) Imaginary part of the complex dielectric spectra (Im ε/M) normalized by molar concentration; (**c**) Absorption coefficients (α/M) normalized by molar concentration, obtained by THz-TDS; and (**d**) Absorption coefficients (α/M) normalized by molar concentration, obtained by FIR for the ionic liquids $[C_6mim^+][Br^-]$, $[C_8mim^+][Br^-]$, and $[C_{10}mim^+][Br^-]$.

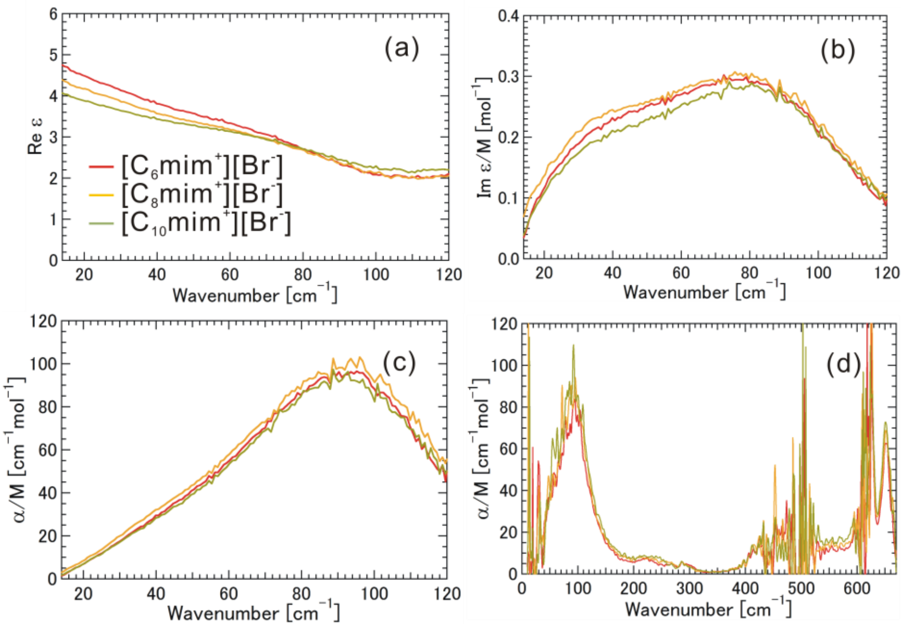

Figure S4. (**a**) Real part of the complex dielectric spectra (Re ε); (**b**) Imaginary part of the complex dielectric spectra (Im ε/M) nomrlized by molar concentration; (**c**) Absorption coefficients (α/M) normalized by molar concentration, obtained by THz-TDS; and (**d**) Absorption coefficients (α/M) normalized by molar concentration, obtained by FIR for the ionic liquids $[C_6mim^+][Cl^-]$ and $[C_8mim^+][Cl^-]$.

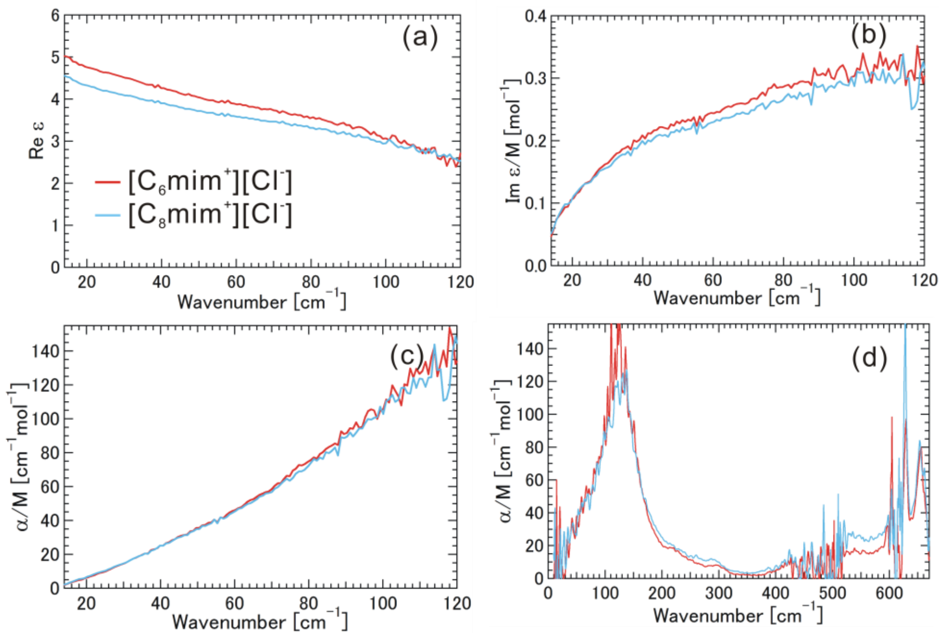

Figure S5. (**a**) Calculated vibrational spectra for the alkyl-methyl-imidazolium cation, [C_6mim$^+$] in the region between 700 and 1000 cm^{-1}; (**b**) Vibrational mode at 746 cm^{-1}; and (**c**) Vibrational mode at 825 cm^{-1}. The absorption bandwidth was set at 10 cm^{-1} for bands to make them easy to see.

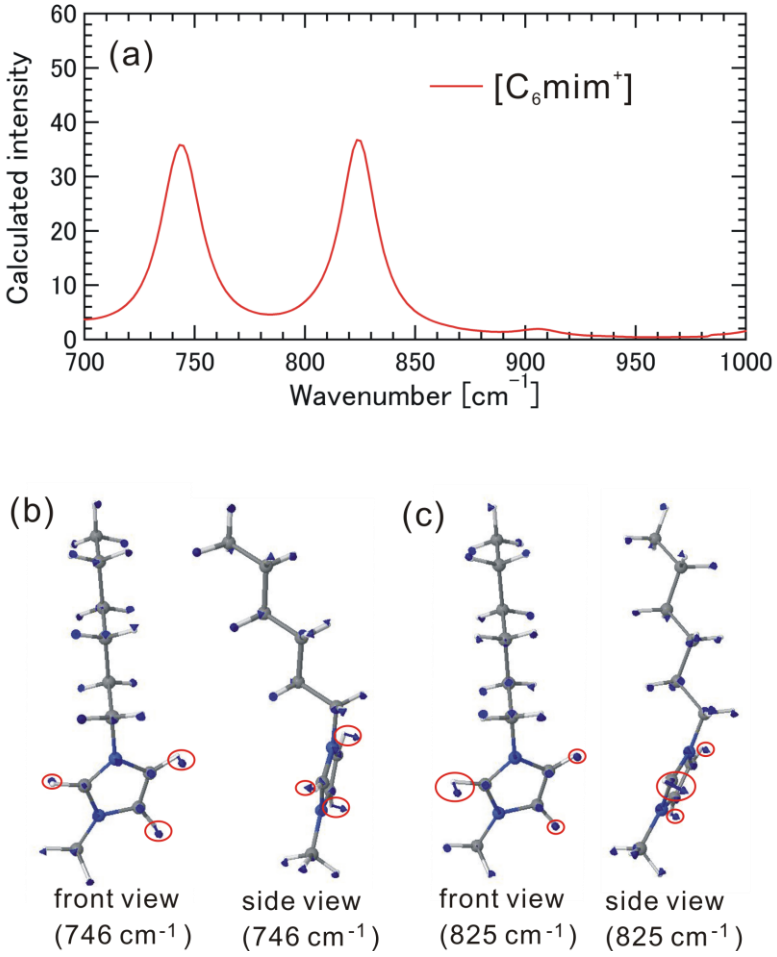

Supplement: Supplementary file 1 [file materials-07-07409-s001.pdf]
